# Organ preservation with total neoadjuvant therapy in early‐stage rectal cancer: A statewide analysis

**DOI:** 10.1111/codi.70446

**Published:** 2026-04-03

**Authors:** Zachary Bunjo, Tracy Fitzsimmons, Thuy‐My Nguyen, Michael Penniment, Sudarshan Selva‐Nayagam, Darren Tonkin, Tiong Cheng Sia, Elizabeth Murphy, Michelle Thomas, Tarik Sammour

**Affiliations:** ^1^ Colorectal Unit, Department of Surgery Royal Adelaide Hospital Adelaide South Australia Australia; ^2^ Discipline of Surgery, Faculty of Health and Medical Sciences, School of Medicine Adelaide University Adelaide South Australia Australia; ^3^ Department of Surgery Lyell McEwin Hospital Elizabeth Vale South Australia Australia; ^4^ Department of Radiation Oncology Royal Adelaide Hospital Adelaide South Australia Australia; ^5^ Department of Medical Oncology Royal Adelaide Hospital Adelaide South Australia Australia; ^6^ Department of Surgery The Queen Elizabeth Hospital Woodville South South Australia Australia; ^7^ Department of Surgery Flinders Medical Centre Bedford Park South Australia Australia

**Keywords:** early‐stage rectal cancer, organ preservation, total neoadjuvant therapy

## Abstract

**Background:**

The standard treatment for early‐stage (cT2‐3N0) rectal adenocarcinoma is upfront Total Mesorectal Excision (TME), but the desire for organ preservation has seen the increasing use of neoadjuvant therapy in these patients. Owing to its likely higher complete response rate, Total Neoadjuvant Therapy (TNT) is an attractive but understudied option. This study aimed to determine outcomes in patients with early‐stage rectal cancer undergoing TNT.

**Methods:**

This was a retrospective study of patients with cT2‐3N0M0 rectal adenocarcinoma who underwent TNT instead of upfront surgery from 2019 to 2025 in South Australia. TNT comprised long‐course chemoradiotherapy followed by up to 8 cycles of FOLFOX or 6 cycles of CAPOX. Patients with cCR were offered watch and wait (W&W) surveillance. The outcomes of interest included cCR rate, regrowth rate, local and distant recurrences (LR and DR) and 3‐year survival.

**Results:**

Thirty‐nine patients were included, with mean tumour distance 4 cm from the anal verge. The median follow‐up was 41 months (IQR 26–61) and 53.8% had completed at least 3 years of follow‐up. Thirty‐one patients (79.5%) achieved a cCR and proceeded to W&W. The regrowth rate was 16.1%; none experienced a LR and three had a DR. The 3‐year DFS in the overall cohort was 94.1% (95% CI 86.3%–100%) and all patients were alive up to 3 years of follow‐up. The rate of Grade 3 or higher chemotherapy‐related toxicity was 25.6% and there were no deaths.

**Conclusion:**

Patients receiving TNT for early‐stage rectal cancer had a very high cCR rate and associated high organ preservation rate. This approach has potential quality of life advantages compared with surgery, with acceptable oncological and toxicity profiles.

**What does this paper add to the literature?**

This paper provides novel data supporting the efficacy of TNT in achieving high rates of clinical complete response and organ preservation in patients with early‐stage rectal cancer, with acceptable oncological and toxicity profiles.

## INTRODUCTION

Despite much attention being directed to advancing treatment in locally‐advanced rectal cancer (LARC) for several decades, the management of early‐stage rectal cancer remains a complex and evolving topic. Whilst local excision may be appropriate for select low‐risk T1 disease, upfront radical resection with Total Mesorectal Excision (TME) remains the standard‐of‐care for most patients with cT2‐3N0 disease [1, 2]. Whilst TME has provided acceptable local recurrence and survival outcomes, such operations are not without risk and carry significant implications for quality of life and the possibility of a permanent stoma depending on tumour height, anastomotic complications and baseline functional status.

Total Neoadjuvant Therapy (TNT) has established itself as the leading treatment for LARC, with high‐quality trial data showing improved disease‐free survival (DFS) and clinical complete response (cCR) rates [3, 4, 5]. Furthermore, non‐operative management with organ preservation has emerged as a feasible option for many of these patients. Perhaps unsurprisingly, this has prompted patients with early‐stage rectal cancer to pursue neoadjuvant therapy, with recent multi‐centre data from the United States demonstrating that over half of patients with early‐stage rectal cancer undergoing proctectomy had received neoadjuvant therapy [6]. Indeed, several trials have been conducted exploring the use of neoadjuvant therapy and local excision (LE) in such patients as an alternative to TME [7, 8, 9]. Whilst delivering promising organ preservation rates, the morbidity of local excision is often under‐recognised [10, 11] and there are some flaws in benchmarking recurrence and survival against TME in a group of patients who are often under‐staged and therefore under‐treated with upfront radical resection, particularly for tumours in the low rectum [12, 13].

With mature watch and wait (W&W) data becoming available for TNT, there is now increasing interest in adopting this treatment strategy for early‐stage rectal cancer patients, particularly those with low tumours who would require a permanent stoma [14]. The potential for higher cCR, organ preservation and better treatment of under‐staged nodal disease makes this an appealing but under‐studied option [15]. Accordingly, the aim of this study was to report response and survival outcomes for patients with early‐stage (cT2‐3N0) rectal adenocarcinoma who had declined standard care radical resection in favour of TNT.

## METHODS

This retrospective study is reported using the Strengthening the Reporting of Observational Studies in Epidemiology (STROBE) statement [16] and received ethics approval from the Central Adelaide Local Health Network Human Research Ethics Committee, St. Andrew's Hospital Research and Ethics Committee, Northern Adelaide Local Health Network Governance Office and Southern Adelaide Local Health Network Governance Office.

### Patient selection

All patients diagnosed with early‐stage (cT2‐3N0M0) rectal adenocarcinoma (without any adverse staging MRI features of extramural vascular invasion or threatened/involved mesorectal fascia) within 15 cm from the anal verge who underwent TNT after declining radical resection from 2019 to 2025 were included from a multi‐institution database in South Australia. This included data from patients treated across five metropolitan hospitals.

### Treatment

The recommended treatment for cT2‐3N0M0 rectal cancer in our setting is upfront radical resection following the principles of TME [17]. For patients with cT2‐3N0 rectal cancer who have declined upfront resection in pursuit of organ preservation, consolidation TNT (cTNT) is typically offered in our setting as an alternative treatment as this likely affords the highest chance of a cCR. cTNT comprises long‐course chemoradiotherapy (LCCRT) administered over 6 weeks, followed by a 2‐week wait period. The total radiotherapy dose is 50–54 Gy with concurrent infusional 5‐FU or oral capecitabine. Subsequently, patients receive consolidation chemotherapy consisting of up to 8 cycles of FOLFOX (over 16 weeks) or 6 cycles of CAPOX (over 18 weeks). In the infrequent instances where induction TNT (iTNT) is chosen for any reason, the regime is the same except for the sequence of LCCRT and chemotherapy being reversed.

### Restaging and subsequent treatment

Patients are restaged with flexible sigmoidoscopy and MRI pelvis 2–4 weeks following the completion of cTNT. The criteria employed for cCR and near‐complete response (nCR) have been described previously [18]. MRI scans were reviewed by radiologists with a particular interest in rectal cancer imaging. MRI protocols utilised included high‐resolution T2‐weighted and Diffusion‐Weighted Imaging (DWI). A radiological cCR was defined as the presence of low‐signal fibrosis on T2 images with a corresponding absence of restricted diffusion on DWI. Conversely, regrowth was defined by the new appearance of intermediate T2 signal or restricted diffusion at the primary tumour bed [19, 20]. Patients achieving a cCR are offered the option of watch and wait (W&W) surveillance, in a protocol consistent with that used in LARC (see Table 1). Patients with a nCR are managed on a case‐by‐case basis and are selectively offered the option of initial W&W (typically limited to reassessment at 3 months) in hope of a conversion to cCR, and radical resection recommended in those who do not.

**TABLE 1:** Watch and wait surveillance protocol.

Rectal exam, flexible sigmoidoscopy, and CEA every 3 months for 1 year, then 6 monthly for 5 years
MRI pelvis every 3 months for 1 year, and then 6 monthly for 5 years
CT chest/abdomen every 12 months for 5 years
Colonoscopy at 1 year (then 6 and 11 years as per usual colorectal cancer surveillance)

### Outcome definitions and recommended management

Regrowth was considered distinct to LR and defined as recurrence of rectal cancer at the local tumour location or regional lymph nodes as demonstrated on endoscopy or imaging, after initially achieving a cCR and prior to undergoing radical resection [21]. Such patients were recommended radical resection. Less commonly, local excision is performed for regrowth limited to the rectal wall; however, this is not standard practice in our setting. Disease‐free survival (DFS) was defined as the time from diagnosis to first LR/DR or death. Overall survival (OS) was defined as the time from diagnosis to death or censored at last follow‐up.

### Data collection and statistical analysis

Data were retrospectively collected from patient electronic medical records. Patient demographics (age and sex), baseline tumour characteristics (cT stage, height above the anal verge on MRI), and TNT characteristics (regime, dose of radiotherapy and chemotherapy agents/cycles and toxicity as per the CTCAE v5.0 [22]) were recorded. Restaging outcomes, regrowth and recurrences, and subsequent management were captured. For patients undergoing surgery, post‐operative complications (graded according to the Clavien‐Dindo classification [23]) and pathological results were documented.

Clinical characteristics and demographics were summarised with frequencies (percentages), mean (standard deviation, SD) or median (interquartile range, IQR). Survival was summarised using Kaplan–Meier curves. Statistical tests were two‐sided, with significance set at *p* < 0.05. Data analysis was performed using IBM SPSS Statistics for Macintosh, Version 31.0 (IBM Corp, Armonk, NY, USA).

## RESULTS

### Patient and treatment characteristics

**FIGURE 1:**
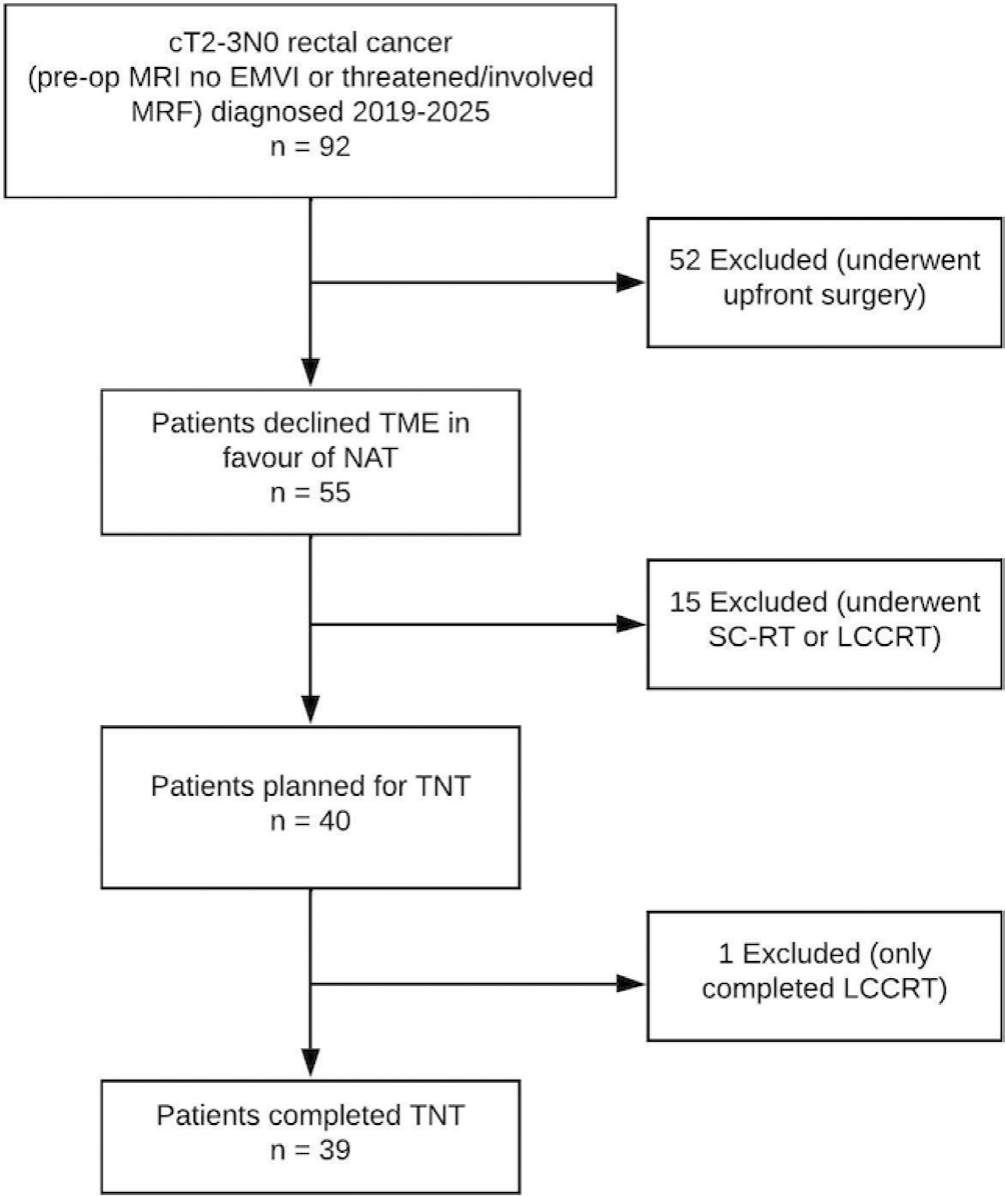
Patient flowchart. EMVI, extramural vascular invasion; LCCRT, long‐course chemoradiotherapy; MRF, mesorectal fascia; NAT, neoadjuvant therapy; SC‐RT, short‐course radiotherapy; TME, total mesorectal excision; TNT, total neoadjuvant therapy.

A total of 39 patients were included (Figure 1). The mean age was 59.3 years (SD 11.7) and 64.1% were male. The mean tumour height was 4.0 cm (SD 2.2) above the anal verge. The proportion of patients with a cT2 tumour was 56.4%, and the remaining 43.6% had a cT3 tumour. All patients received cTNT aside from one who received iTNT. All patients received at least 50 Gy of radiotherapy. For patients receiving FOLFOX, the median cycles of chemotherapy received were 7.5 cycles (range 2–8 cycles) and 87.5% received at least 4 cycles. For patients receiving CAPOX, the median cycles of chemotherapy received were 6 cycles (range 1–6 cycles) and 96.8% received at least 3 cycles. Ten patients (25.6%) had a Grade 3 or greater chemotherapy‐related toxicity, the most common of which were nausea/vomiting and diarrhoea. There were no treatment‐related deaths. The median follow‐up was 41 months (IQR 26–61) and 53.8% had completed at least 3 years of follow‐up at the time of analysis.

### Response and regrowth characteristics

**FIGURE 2:**
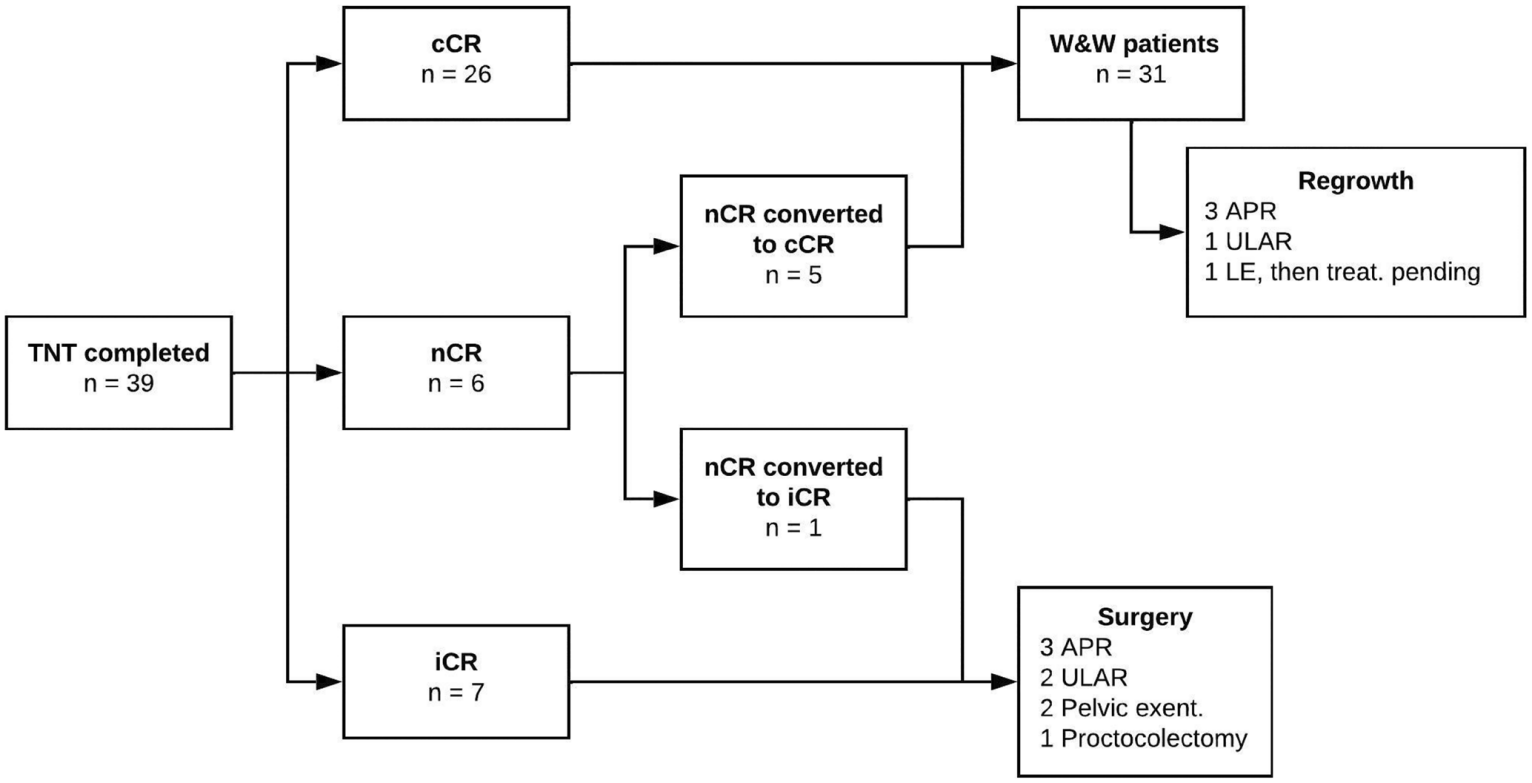
Summary flow of treatment responses and subsequent management. APR, abdominoperineal resection; cCR, clinical complete response; iCR, incomplete clinical response; LE, local excision; nCR, near‐complete response; TNT, total neoadjuvant therapy; ULAR, ultra‐low anterior resection; W&W, watch and wait.

Thirty‐one patients (79.5%) achieved a cCR, all of whom proceeded to W&W surveillance. A flowchart of response types and subsequent treatment is shown in Figure 2. At the time of analysis, 26 patients (66.7%) in the overall group had sustained organ preservation.

Regrowth, or nCR becoming an iCR, occurred in 15.4% at a median of 9 months (IQR 6–12) following completion of TNT. The characteristics of regrowths are shown in Table 2. The latest regrowth after cCR occurred at 35 months. One patient declined TME in favour of local excision for mucosal regrowth, and subsequently developed further regrowth, the treatment of which is pending. Among the 12 patients who underwent TME for either regrowth or iCR, the R0 resection rate was 91.7% and one patient had a pCR (8.3%). For those patients who had not achieved a pCR, pathology revealed pT1N0 disease in 2 patients (18.2%), pT2N0 disease in 4 patients (36.4%), pT3N0 disease in 4 patients (36.4%) and pT3N1 disease in 1 patient (9.1%). The overall permanent stoma rate was 69.2%. It should be noted that one patient with a regrowth underwent a posterior exenteration (abdominoperineal resection with posterior vaginectomy) due to anterior tumour location with no clear plane to the posterior vaginal wall. The other exenteration case was a patient requiring a total pelvic exenteration due to an iCR with fibrosis extending towards the prostate with no clear margin and concern for disease involvement. There were no 30‐day post‐operative mortalities.

**TABLE 2:** Characteristics of regrowths and local recurrences.

Patient	Sex	Age (years)	Tumour characteristics	TNT regime	Nature of regrowth	Time of regrowth since cCR (months)	Local recurrence	Current status
1	Male	38	cT3, 6 cm FAV	LCCRT (54 Gy, 27#) + 6 cycles CAPOX	Mucosal	13	No	Alive and disease free (85 months)
2	Male	62	cT3, 5 cm FAV	LCCRT (50 Gy, 25#) + 6 cycles CAPOX	Rectal wall + nodal	10	No	Died of CRC (48 months)
3	Male	53	cT3, 3 cm FAV	LCCRT (50 Gy, 25#) + 4 cycles CAPOX	Mucosal	5	No	Alive and disease free (47 months)
4	Male	52	cT2, 5 cm FAV	LCCRT (50 Gy, 25#) + 6 cycles CAPOX	Mucosal (treated with LE) followed by mucosal + mesorectal regrowth (13 months later)	8	No	Alive with disease (31 months, awaiting TME)
5	Female	60	cT2, 2 cm FAV	LCCRT (50 Gy, 25#) + 2 cycles FOLFOX	Mucosal	35	No	Alive and disease free (56 months)

Abbreviations: #, fractions; cCR, clinical complete response; FAV, from anal verge; Gy, Gray; LCCRT, long‐course chemoradiotherapy; LE, local excision; TME, total mesorectal excision; TNT, total neoadjuvant therapy.

### Oncological outcomes

**FIGURE 3:**
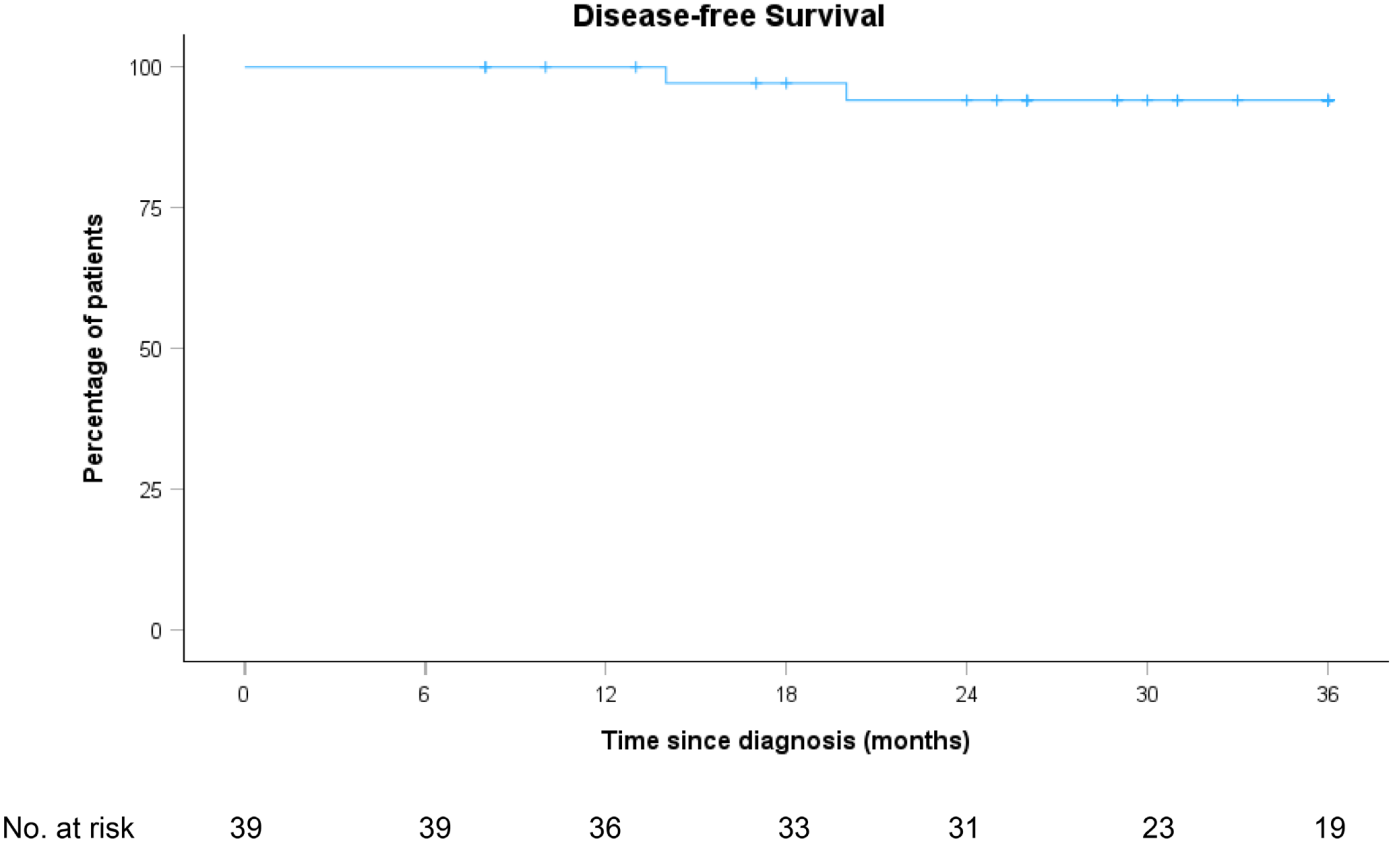
Overall group 3‐year DFS.

The 3‐year DFS (Figure 3) in the overall cohort was 94.1% (95% CI 86.3–100%). The 3‐year DFS stratified by response groups is shown in Table 3. All patients were alive up to 3 years of follow‐up, with one death at 48 months of metastatic rectal cancer. No patients had an LR, and three patients had a DR (two within 20 months and one at 37 months). Details of the three patients who had a DR are summarised in Table 4. It should be noted that Patient 3 had initially declined surgery after TNT, resulting in an almost 6‐month delay to surgery.

**TABLE 3:** Disease‐free survival by response group.

Group	Median follow‐up (months)	3‐year DFS
Sustained cCR (*n* = 26)	39 (IQR 23–61)	100%
Regrowth *or* nCR converted to iCR (*n* = 6)	48 (IQR 33–63)	83.3% (95% CI 53.5%–100%)
Initial iCR (*n* = 7)	31 (IQR 26–64)	85.7% (95% CI 59.8%–100%)

Abbreviations: cCR, clinical complete response; DFS, disease‐free survival; iCR, incomplete clinical response; nCR, near‐complete response.

**TABLE 4:** Characteristics of patients with distant recurrence.

Patient	Sex	Age (years)	Tumour characteristics	TNT regime	Initial outcome	Regrowth and subsequent management	Recurrence	Current status
1	Male	38	cT3, 6 cm FAV	LCCRT (54 Gy, 27#) + 6 cycles CAPOX	cCR → W&W	Yes (13 months), underwent ULAR (pT3N0)	DR (20 months), lung (resected)	Alive and disease free (85 months)
2	Male	62	cT3, 5 cm FAV	LCCRT (50 Gy, 25#) + 6 cycles CAPOX	cCR → W&W	Yes (10 months), underwent APR (pT3N0)	DR (37 months), liver (resected and recurred)	Died of CRC (48 months)
3	Male	68	cT2, 4 cm FAV	LCCRT (50 Gy, 25#) + 6 cycles CAPOX	iCR, patient delayed surgery, proceeded to TPE (pT2N0)	N/A	DR (14 months), lung (SABR)	Alive and disease free (48 months)

Abbreviations: #, fractions; APR, abdominoperineal resection; cCR, clinical complete response; CRC, colorectal cancer; DR, distant recurrence; FAV, from anal verge; Gy, Gray; LCCRT, long‐course chemoradiotherapy; nCR, near‐complete response; SABR, stereotactic ablative body radiotherapy.; TNT, total neoadjuvant therapy; TPE, total pelvic exenteration; ULAR, ultra‐low anterior resection; W&W, watch and wait.

## DISCUSSION

This study is among the earliest reporting outcomes for a cohort of patients with early‐stage (cT2‐T3N0) rectal cancer declining standard care upfront radical resection in favour of TNT. Notably, there was a high cCR rate of almost 80%, allowing these patients to pursue organ preservation with W&W surveillance. Acknowledging variable follow‐up periods, the 3‐year DFS and OS were high at 94.1% and 100%, respectively, with an acceptable toxicity profile.

The treatment of early‐stage rectal cancer is evolving, and neoadjuvant approaches are increasingly being investigated [14]. Multi‐centre data from the United States has shown that over half of early‐stage patients undergoing proctectomy had received neoadjuvant treatment [6] which is likely driven by an increasing emphasis on pursuing non‐operative management and organ preservation. This has seen the greatest uptake in patients with low rectal tumours facing the prospect of an abdominoperineal resection and permanent colostomy and is also reflected in this study where the mean tumour height was 4 cm. In 2019, Habr‐Gama's group demonstrated a 30% cCR rate in T2N0 low rectal cancers undergoing standard chemoradiotherapy (CRT), which increased to 67% with extended CRT (radiation dose‐escalation and added consolidation chemotherapy) [24]. The main appeal of adopting TNT in early‐stage rectal cancer is the potential for increased cCR compared to conventional chemoradiotherapy, as has been suggested by the aforementioned work by Habr‐Gama and trial data in LARC [5, 25, 26, 27]. There is scant literature reporting the use of TNT in early‐stage rectal cancer. A recently published series documented 16 patients with stage I rectal cancer receiving either upfront or post local excision consolidation TNT instead of TME as would be standard [15]. The overall complete response rate was 93.7% and organ preservation rate was 87.5%. In contrast to our study, lower‐risk T1 tumours were included and there was better TNT compliance, which likely explains the higher complete response and organ preservation rate despite a similar consolidation TNT regime. Beyond retrospective series, several trials are currently underway investigating the efficacy of TNT in early‐stage rectal cancer [14, 28]. These trials focus on a similar population to this study of cT2‐3 N0 disease where upfront TME represents standard care.

Beyond organ preservation, TNT for early‐stage rectal cancer may have a survival advantage. Clinical under‐staging is a known issue in early rectal cancer, with studies indicating a nodal upstaging rate of around 20% following upfront surgery, with mixed data on the effectiveness of adjuvant chemotherapy in upstaged patients [12, 13, 29]. Low tumours have the additional issue of disproportionately higher lateral nodal involvement owing to their preferential lymphatic drainage to the lateral compartment [30], which is not routinely dissected or treated in the West. As such, given low tumours are particularly prone to adverse upstaging, the disposition towards organ preservation with TNT in these patients makes even more sense.

Numerous trials have explored organ preservation in early rectal cancer, by using conventional chemoradiotherapy combined with LE. A meta‐analysis that compiled such studies and compared this with TME demonstrated an organ preservation rate of 75.4% at mean follow‐up of 5.6 years with no significant difference in DFS, OS or LR [31]. The recently published TAUTEM trial comprised a similar population of cT2‐3N0 rectal cancer patients randomised to either TME or chemoradiotherapy followed by LE [7]. The authors found comparable recurrence and survival outcomes, paired with an organ preservation of 77.8% in the modified intention‐to‐treat analysis. Looking at a comparable group of cT2‐3 N0 rectal cancer patients included in the TAUTEM trial, the standard approach of upfront TME afforded patients a 2‐year OS of 85.2%. However, this is perhaps lower than would be expected in the modern era where a 90–95% 3‐year OS is seen in LARC with TNT, and International Watch & Wait Database (IWWD) data of all‐comer rectal cancer patients undergoing W&W indicating an 85% 5‐year OS [21]. This highlights a limitation in benchmarking radiotherapy and local excision‐based treatments against TME in early‐stage rectal cancer and may give further merit to the use of TNT, which in this albeit small study, produced 3‐year DFS and OS well over 90%.

There are also some unique considerations when looking at outcomes in rectal cancer patients undergoing W&W, which form a large proportion of this study group. The IWWD study revealed a 25.2% local regrowth rate in all‐comer rectal cancer patients, the majority of which occurred in the first 2 years [21]. Considering recurrences more broadly, trials of cT2/3 patients undergoing neoadjuvant therapy with LE have shown LR and DR rates of 6.2–11% and 4–12.3%, respectively [7, 8, 32]. In this study of cT2‐3 N0 tumours, regrowth occurred in 16.1% at latest 35 months following the completion of TNT. Distant recurrence occurred in three patients, all of whom had either experienced a regrowth or had an iCR following TNT. This observation may lead to the concern that delayed treatment of residual local disease may increase the risk of distant recurrence; however, surgery occurred within 13 months in each of these patients (one patient self‐delayed surgery) with subsequent favourable node‐negative pathology. The more plausible explanation would be that distant failure resulted in the context of biologically higher‐risk disease that also contributed to these patients developing local regrowth. Had these patients undergone standard care upfront surgery, they may have still developed distant metastases. Salvage surgery has been shown to be successful in both early rectal and all‐comer populations undergoing W&W following neoadjuvant therapy [33, 34, 35].

Another concern regarding the use of TNT in early‐stage disease is treatment toxicity and ‘over‐treatment’. Around a quarter of patients in our study had Grade 3 or 4 chemotherapy‐related toxicity, which broadly aligns with data from LARC TNT patients [5, 36, 37] but contrasts with the previously discussed stage I rectal cancer TNT series by Erozkan et al. which reported full treatment compliance without any toxicity, with a very similar TNT regime of LCCRT followed by 8 cycles FOLFOX/5 cycles CAPOX [15]. This is difficult to explain beyond the possibility that the inclusion of earlier, lower‐risk tumours in that study may have included less comorbid patients. Whilst the use of only radiotherapy has appeal in mitigating chemotherapy toxicity, this likely comes at the cost of lower cCR rates and poorer survival in under‐staged patients. Nevertheless, this underscores the importance of further refining TNT regimes in the early rectal cancer context to strike the optimal balance between enhanced organ preservation and oncological outcomes with acceptable treatment toxicity. Indeed, this is being tested in the currently recruiting EARLY‐TNT Trial, whereby a response assessment halfway through consolidation chemotherapy affords early responders the opportunity to avoid additional chemotherapy and thereby potentially reduce toxicity [38]. Along similar lines, quality of life (QoL) and functional outcomes are also crucially important. Data have demonstrated that LARC patients managed non‐operatively with W&W following TNT may have improved QoL with much lower bowel and bladder dysfunction compared to conventional treatment [39] which has also been corroborated in early rectal cancers treated with other neoadjuvant therapy approaches [40, 41, 42]. Indeed, it is no longer clear to many whether upfront surgery may ultimately be considered ‘over‐treatment’ in this patient cohort, as it is in anal squamous cell carcinoma, for example.

Limitations of this study include its retrospective design and small sample size with variable follow‐up intervals. The inclusion of patients with shorter follow‐up introduces the potential for events to be missed; however, given the majority of regrowths and recurrences occurred early (i.e., within 3 years), this is unlikely to be a significant issue in this study. Given the population comprised patients declining recommended surgery in favour of TNT, an element of selection bias must also be considered in result interpretation. The preponderance of lower tumours limits the applicability of the study findings to all‐comers with early‐stage rectal cancer. Further validation of TNT in early‐stage rectal cancer is required through prospective clinical trials, such as EARLY‐TNT [38].

In conclusion, the use of TNT in cT2‐3 N0 rectal cancer appears to have high efficacy in achieving cCR and allowing organ preservation, and potentially very high survival and acceptable toxicity profiles. Although further validation is required, including a more robust comparison against other neoadjuvant therapy and surgical approaches, the findings of this study would suggest that in selected early‐stage rectal cancer patients declining standard care upfront radical resection, TNT can be considered and discussed as an alternative treatment option.

## AUTHOR CONTRIBUTIONS

**Zachary Bunjo:** Writing – original draft; writing – review and editing; conceptualization; methodology; investigation; data curation; formal analysis. **Darren Tonkin:** Writing – review and editing; methodology; data curation. **Elizabeth Murphy:** Methodology; writing – review and editing; data curation. **Michelle Thomas:** Supervision; writing – review and editing; methodology.

## FUNDING INFORMATION

Zachary Bunjo is supported by the Clinician PhD Pathway Central Adelaide Local Health Network in collaboration with The University of Adelaide, with funds by the Health Services Charitable Gifts Board.

## CONFLICT OF INTEREST STATEMENT

No conflicts of interest exist.

## ETHICS STATEMENT

Received ethics approval from the Central Adelaide Local Health Network Human Research Ethics Committee (HREC/15/RAH/186), St. Andrew's Hospital Research and Ethics Committee (#117), Northern Adelaide Local Health Network Governance Office and Southern Adelaide Local Health Network Governance Office. Waiver of patient consent per ethics approval. Material from other sources not reproduced.

## Supporting information


Data S1:

## Data Availability

Research data are not shared.
